# Inborn errors of thymic stromal cell development and function

**DOI:** 10.1007/s00281-020-00826-9

**Published:** 2020-11-30

**Authors:** Alexandra Y. Kreins, Stefano Maio, Fatima Dhalla

**Affiliations:** 1 UCL Great Ormond Street Institute of Child Health, London, UK; 2 Department of Immunology, Great Ormond Street Hospital for Children NHS Foundation Trust, London, UK; 3 Developmental Immunology, Weatherall Institute of Molecular Medicine, University of Oxford, Oxford, UK; 4 Department of Clinical Immunology, Oxford University Hospitals, Oxford, UK

**Keywords:** Thymus, Thymic stromal cells, Immunodeficiency, DiGeorge syndrome, FOXN1, Thymus transplantation

## Abstract

As the primary site for T cell development, the thymus is responsible for the production and selection of a functional, yet self-tolerant T cell repertoire. This critically depends on thymic stromal cells, derived from the pharyngeal apparatus during embryogenesis. Thymic epithelial cells, mesenchymal and vascular elements together form the unique and highly specialised microenvironment required to support all aspects of thymopoiesis and T cell central tolerance induction. Although rare, inborn errors of thymic stromal cells constitute a clinically important group of conditions because their immunological consequences, which include autoimmune disease and T cell immunodeficiency, can be life-threatening if unrecognised and untreated. In this review, we describe the molecular and environmental aetiologies of the thymic stromal cell defects known to cause disease in humans, placing particular emphasis on those with a propensity to cause thymic hypoplasia or aplasia and consequently severe congenital immunodeficiency. We discuss the principles underpinning their diagnosis and management, including the use of novel tools to aid in their identification and strategies for curative treatment, principally transplantation of allogeneic thymus tissue.

## Introduction

The thymus is the primary lymphoid organ responsible for the generation and selection of a T cell repertoire able to respond to foreign antigens whilst remaining tolerant to self [1].

In terms of cellular composition, the thymus is mainly comprised of developing thymocytes [2, 3]. It is, however, structurally and functionally dependent on rare stromal cell populations, the main constituent of which are endodermally derived thymic epithelial cells (TEC) [2, 4]. TEC form a specialised three-dimensional scaffold and can be categorised into two main lineages, cortical (c-) and medullary (m-) TEC, based on anatomical, molecular and functional characteristics [5–7]. Aside from TEC, the thymic stroma includes mesenchymal and vascular elements. As a composite, these stromal cells form a unique microenvironment enabling homing of lymphoid progenitors to the thymus, where TEC support their commitment to a T cell fate, their differentiation to functional maturity and the selection of a T cell receptor (TCR) repertoire bespoke for an individual organism [5–7]. Mesenchymal cells originating from the neural crest ectoderm contribute to the thymic capsule, septae, fibroblasts and pericytes, whereas those with mesodermal origin give rise to endothelial cells [2, 3, 8]. Collectively, mesenchymal cells play important roles in the early stages of thymic organogenesis and later promote thymocyte trafficking and provide growth factors for TEC [2, 3]. Other haematopoietic cells also contribute to thymic cellularity, including dendritic cells (DCs), macrophages and B cells, with roles in antigen presentation, thymocyte selection and clearance of apoptotic thymocytes [3].

During human embryogenesis, the thymus and inferior parathyroids develop from the 3rd pharyngeal pouches (PP), out-pouchings of endodermal foregut, in a process that begins by early in week 6 of gestation [9, 10]. Formation and early patterning of the 3rd PP are under the control of a number of transcription factors, including HOXA3, EYA1, PAX1 and -9, SIX1 and -4, and TBX1 [10–16]. Initially, the thymic and parathyroid primordia occupy a common domain, surrounded by a mesenchymal capsule derived from the neural crest ectoderm [10]. Between gestational weeks 7 and 9 the two thymic domains migrate medially and caudally, becoming separated from the parathyroid domains and eventually meeting to form a bilobed organ in the anterior mediastinum [9, 10]. *FOXN1*, the master transcriptional regulator of TEC, is detectable in the thymic domains within the 3rd PPs from midweek 6 [10, 17, 18]. At this stage, TEC have an undifferentiated phenotype and contain bipotent progenitor cells, which subsequently differentiate into c- and m-TEC, in a FOXN1-dependent manner, starting from midweek 8 [10, 19, 20]. At the same time, the first T-lineage precursors are detected, with their frequency increasing dramatically from the 10th week of gestation following the establishment of the vasculature [10]. The developmental programmes of TEC and thymocytes unravel in parallel and are highly interdependent. Bidirectional lympho-stromal interactions, designated “crosstalk”, are critical for thymic organogenesis, TEC differentiation, maturation, organisation, function and postnatal maintenance, and consequently proper thymopoiesis [5–7, 21, 22]. The induction of the thymic cortical microenvironment relies on the presence of early T cell progenitors [23]. The mTEC compartment is further dependent on crosstalk signals from the tumour necrosis factor superfamily of molecules, including receptor activator of NFκB (RANK), CD40 and lymphotoxin-β, which activate cell surface receptors expressed on TEC [21, 24]. Intrathymic lymphoid cells, principally single positive αβ thymocytes that have recently undergone positive selection by cTEC, provide their ligands to induce mTEC development and functional maturation, culminating in the expression of the autoimmune regulator (AIRE) and the costimulatory molecules necessary for T cell central tolerance induction [21, 24].

Inborn errors of thymic stromal cells are a rare but important group of pathologies. Aberrations in: (1) 3rd PP patterning and thymic organogenesis, (2) TEC development, due to TEC-intrinsic defects or secondary to impaired lympho-stromal crosstalk and (3) TEC function, are implicated in the pathogenesis of human thymic stromal cell defects. The indispensable role that the thymic stroma plays in T cell lymphopoiesis and repertoire selection is reflected in the immunological consequences of these conditions, namely T cell immunodeficiency and/or early onset autoimmunity [25]. The most serious clinical expression of a thymic stromal cell defect is profound T cell lymphopaenia, presenting as a complete DiGeorge syndrome or severe combined immune deficiency (T^-/low^B^+^NK^+^ SCID). Such cases present early in infancy with severe, life-threatening infections. The spectrum of infections is broad, including viral, bacterial, opportunistic and vaccine-related infections, reflecting absent/severely impaired cell–mediated immunity and consequently impaired help for antibody production. This may evolve into Omenn syndrome (OS), which is characterised by generalized erythroderma, chronic diarrhoea, failure-to-thrive, lymphadenopathy, hepatosplenomegaly, eosinophilia, elevated IgE and oligoclonal expansions of peripheral T cells [26, 27]. OS should be differentiated from materno-fetal graft-versus-host disease (GVHD) due to engraftment of maternal T cells, which presents with a similar phenotype. A low index of suspicion is essential to facilitate prompt recognition and allow early institution of appropriate interventions to optimise patient outcomes. Importantly, inborn errors of thymic stromal cells need to be differentiated from primary haematopoietic cell defects as therapeutic approaches differ.

**Fig. 1:**
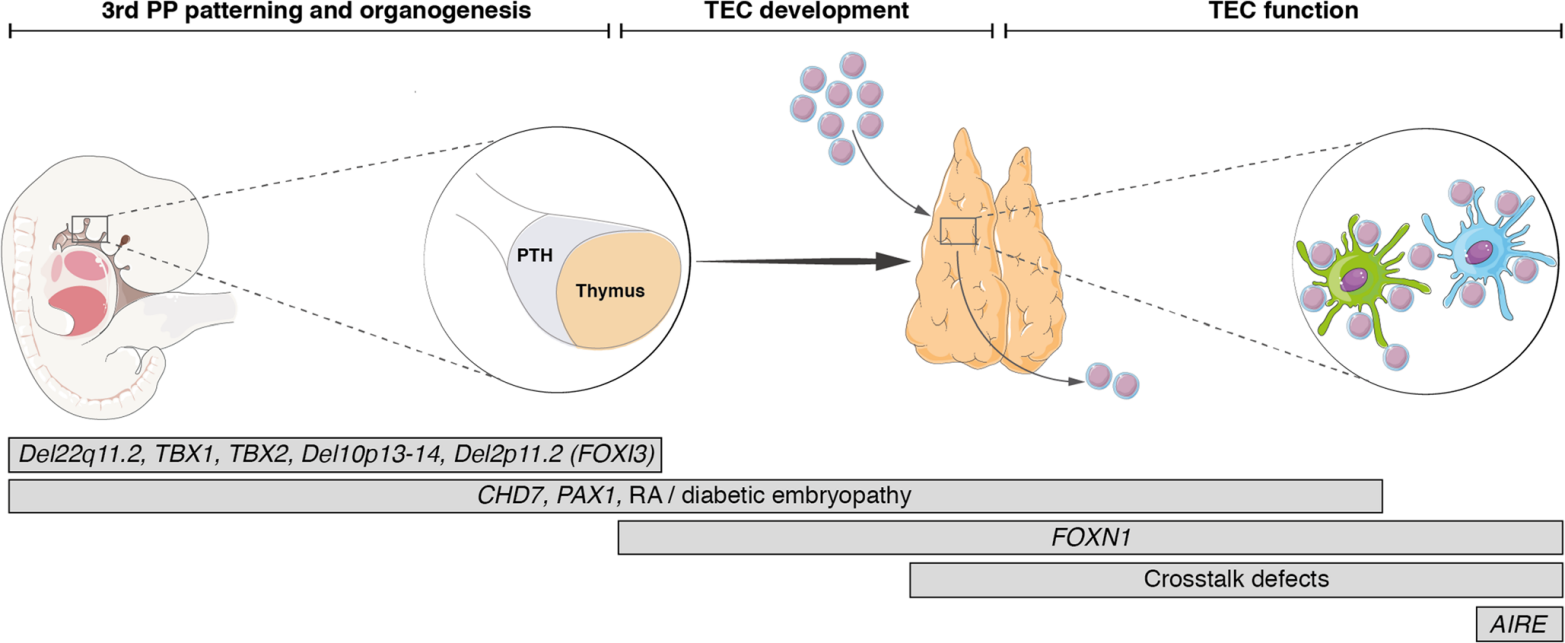
The known inborn errors of thymic stromal cells and their relative effects on 3rd pharyngeal pouch patterning, thymic organogenesis, thymic epithelial cell development and function. PP = pharyngeal pouch; PTH = parathyroids; RA = retinoic acid; TEC = thymic epithelial cell

In the sections that follow, and summarised in Fig. 1 and Table 1, we consider the thymic stromal cell defects described in humans to date, with an emphasis on those causing predominant immune deficiency. We also discuss some common principles to guide their investigation and management, focussing on allogeneic thymus transplantation.

**Table 1:** Genetic associations of inborn errors of thymic stromal cells. References are cited within the main text

Condition	Genetic defect	Inheritance	Other names	Immunological features	Associated features
22q11 deletion syndrome	*Del22q11.2*	De novo (90–95%) AD (5-10%)	DGS Velocardiofacial syndrome	• Variable DGS features*: • cDGS +/- atypical features/OS • pDGS • Mild T lymphopaenia with improvement over time • T cell proliferation may be impaired • Thymus: present/normal, hypoplastic or absent • Ab production may be impaired • Can be asymptomatic • Immunology may be normal • Autoimmunity, atopy	• CHD • Hypoparathyroidism • Other endocrinopathies • Facial dysmorphism • Palatal abnormalities • Feeding difficulties • Genitourinary abnormalities • Developmental delay • Psychiatric disorders
TBX1 deficiency	*TBX1*	AD		• Variable DGS features (see above)*	• CHD • Hypoparathyroidism • Other endocrinopathies • Facial dysmorphism • Palatal abnormalities • Deafness • Psycho-developmental abnormalities
TBX2 deficiency	*TBX2*	AD		• Variable DGS features (see above)*	• Craniofacial dysmorphism • CHD • Hypoparathyroidism • Other endocrinopathies • Cleft lip/palate • Skeletal malformations • Developmental delay
Partial monosomy 10p	*Del10p13-14*	De novo (1 familial deletion reported)	HDR DGS2	• Variable DGS features (see above)*	• Hypoparathyroidism • Sensorineural hearing loss • Renal anomalies • Craniofacial dysmorphism • Developmental delay • Growth retardation
CHARGE syndrome	*CHD7* *(?SEMA3E)*	Most de novo		• Variable DGS features (see above)*	• Coloboma • CHD • Atresia choanae • Retarded growth/development • Genital hypoplasia • Ear anomalies/deafness • Cranial nerve dysfunction • Feeding difficulties • Anosmia • Trachea-oesophageal anomalies • Hypoparathyroidism
2p11.2 microdeletions	*Del2p11.2*	AD (4/5 kindreds) De novo (1/5 kindreds)		• Variable DGS features (see above)*	• (Transient) hypocalcaemia • Asymmetric crying face • Skeletal and palatal abnormalities (1 patient) • Hearing impairment
FOXN1 deficiency	*FOXN1*	AR	Nude SCID	• T^-/low^B^+^NK^+^ SCID +/- OS • Athymia • Absent/severely naïve T cells, RTEs, TRECs • Impaired T cell proliferation • Impaired Ab production	• Congenital alopecia totalis • Nail dystrophy
	*FOXN1*	CH AD	Hypomorphic FOXN1 deficiency	• Wide spectrum: T^-/low^B^+^NK^+^ SCID to mild T cell lymphopaenia with improvement over time • Low TRECs • Can be asymptomatic	• +/-Nail dystrophy
OTFCS2	*PAX1*	AR		• T^-/^B^+^NK^+^ SCID +/- OS • Athymia • Absent/severely naïve T cells, RTEs, TRECs • Impaired T cell proliferation • Impaired Ab production	• Facial dysmorphism • Ear anomalies, preauricular pits, deafness • Branchial cysts/fistulas • Vertebral & shoulder girdle malformations • Mild intellectual impairment
EXTL3 deficiency	*EXTL3*	AR	Immunoskeletal dysplasia with neuro-developmental abnormalities	• Variable • T^-^B^+^NK^+^SCID, +/- OS • Milder T cell lymphopaenia with improvement over time • Or normal immunology	• Short limbed skeletal dysplasia: platyspondyly, brachydactyly, short stature, kyphoscoliosis, cervical spinal stenosis, pelvic anomalies. • Craniofacial dysmorphism • Motor delay, hypotonia, seizures • Hepatic cysts
AIRE deficiency	*AIRE*	AR	APS1 APECED	• Early onset multisystem autoimmunity • Classical triad = CMC, hypoparathyroidism, Addison’s • Auto-Abs including anticytokine Abs • Asplenia	• Ectodermal dystrophy
	*AIRE*	AD		• Delayed onset, attenuated autoimmune phenotypes, incomplete penetrance	• Ectodermal dystrophy

*Ab* Antibody; *AD* autosomal dominant; *APECED* autoimmune polyendocrinopathy candidiasis ectodermal dystrophy; *APS1* autoimmune polyglandular syndrome 1; *AR* autosomal recessive; *CH* compound heterozygous; *CHD* congenital heart disease; *CMC* chronic mucocutaneous candidiasis; *DGS* DiGeorge syndrome; *cDGS* complete DGS; *pDGS* partial DGS; *OS* Omenn syndrome; *OTFCS2* otofaciocervical syndrome type 2; *RTEs* recent thymic emigrants; *SCID* severe combined immune deficiency; *TRECs* T cell receptor excision circles.

## DiGeorge syndrome

DiGeorge syndrome (DGS) is characterised by a triad of thymic hypoplasia, congenital heart disease (CHD) and hypoparathyroidism, often additionally associated with typical dysmorphic facies [28–30]. The cooccurrence of these features is based on the connected embryogenesis of the affected structures.

The most frequent molecular aetiology for DGS is chromosome 22q11.2 deletion syndrome (22q11.2del) [30, 31]. The terms DGS and 22q11.2del are often used interchangeably, which can be a source of confusion particularly as a number of other genetic and environmental factors have been identified in syndromic patients with features of DGS. Most common amongst these are mutations in *CHD7*, causing CHARGE syndrome and maternal diabetes. However, there are several even rarer genetic associations of DGS, as well as a links to other foetal toxins. Therefore the historical terminology, “DGS”, is best used for patients with undefined causality [32] or in reference to a heterogeneous group of disorders sharing hallmark phenotypic features that indicate aberrant patterning of the pharyngeal apparatus.

DGS is associated with a wide spectrum of immunodeficiency [30, 33]. A minority of patients suffer from thymic aplasia, i.e., a complete absence of thymic tissue, with absolute T cell counts below the 10th percentile for age and a naïve CD4^+^ cell count of less than 50 × 10^6^/L [34]. Such patients with complete DGS (cDGS) require definitive management to correct the underlying immunodeficiency for their survival. By contrast, infants with partial DGS (pDGS) have T cell counts below the 10th percentile, but naïve CD4^+^ cells of more than 50 × 10^6^/L. Patients with pDGS and those with milder forms of T cell lymphopaenia require supportive therapies only. Their total T cell counts may improve over time due to lymphopaenia-induced homeostatic expansion, which is associated with oligoclonal expansion of T cells with a memory phenotype (i.e. CD45RO^+^) [30, 35] and abnormal TCR spectratypes [36–38].

## Chromosome 22q11.2 deletion syndrome

22q11.2del is the most common microdeletion syndrome in humans, affecting approximately 0.25 in 1000 births [39–42]. The clinical consequences are highly variable and its actual incidence is likely higher [43, 44]. Chromosome 22q.11.2 deletions are found in over 90% of DGS patients [30]. Most occur de novo [43], reflecting a high rate of spontaneous deletions in this region, which contains four blocks of low copy repeats (LCR), called LCR22A-D. During meiosis, sequence homology between LCRs can lead to misalignment of homologous chromosomes with subsequent non-allelic homologous recombination (NAHR) [45–47]. This results in genomic instability with both deletions and duplications occurring. In most patients (89%), NAHR between LCR22A and LCR22D mediates a large deletion of approximately 3 Mb leading to haploinsufficiency of > 100 genes. A less frequent deletion (5–8%) of 1.5 Mb spans LCR22A and LCR22B encompassing 30 genes. Both deletions include the key gene, *TBX1*. In rare cases, deletions are generated between the other LCRs. Deletions that do not encompass *TBX1* are associated with better T cell counts, but overall, there is no correlation between the size of the deletion and the clinical phenotype [48]. The deletions have variable breakpoints and comprise different genes, but the absence of genotype-phenotype correlation is striking with variable clinical penetrance between individuals with inherited deletions and discordant phenotypes in monozygotic twins [43, 49].

Clinical problems often include CHD, facial dysmorphism and palatal anomalies, hypoparathyroidism and immunodeficiency [30, 43, 44]. Cardiac anomalies occur in more than 50% of patients and are the most common cause of death [50]. These typically affect the cardiac outflow tract causing Tetralogy of Fallot, interrupted aortic arch, ventriculoseptal defect or truncus arteriosus. Approximately half suffer from hypoparathyroidism, with variable clinical manifestations due to hypocalcaemia, ranging from seizures to chronic fatigue and feeding difficulties [51]. The risk of hypocalcaemia can be exacerbated by intercurrent infections or trauma, as well as by transiently increased calcium requirements, for example during adolescence and pregnancy [52, 53]. Over time, other clinical issues may emerge, including developmental delay, psychiatric illness, renal insufficiency, atopy, autoimmunity and malignancy [29].

Immunodeficiency is common in 22q11.2del with some degree of T cell lymphopaenia in 75–80% of patients. Like the other features, there is a wide spectrum of severity, which correlates with variability in thymus size [54]. In most cases, T lymphopaenia is moderate with average absolute T cell counts approximately half those of healthy infants [30, 35]. Children with mild-moderate T lymphopaenia have recurrent respiratory viral infections, often aggravated by secondary bacterial infections. The contribution of nonimmunological factors such as anatomical anomalies, allergies and gastroesophageal reflux to infection susceptibility is significant [30, 55]. In healthy children, thymic involution begins in the second year of life and peripheral T cell counts decline as a consequence. As this occurs, T cell counts of 22q11.2del patients and healthy controls converge [30, 56], and adults with 22q11.2del often have normal total T cell counts [35, 57]. This is partly due to homeostatic proliferation of peripheral T cells, which further impinges on TCR diversity and contributes to T cell exhaustion, both factors which may impair T cell function despite normal numbers. B cell dysregulation with antibody deficits and poor vaccine responses have increasingly been reported in older patients, likely secondary to impaired T cell support [57, 58]. Additionally, autoimmunity is significantly increased [30, 44], particularly in the context of homeostatic expansion of self-reactive T cells and/or T regulatory cell deficiency [33, 59]. A small number of 22q11.2del patients (< 1%) suffer from cDGS [43] and require thymus transplantation or a fully matched haematopoietic stem cell transplant (HSCT) in the first 2 years of life [34]. No reliable predictors for severe immunodeficiency in 22q11.2del have been identified. No association was found between the complexity of the cardiac defect and the severity of the immune deficiency [60]. It has been suggested that the presence of hypoparathyroidism might correlate with thymic involvement due to their intimately related developmental origins [61]; however, the predictive value of hypocalcaemia is not absolute. Therefore, in the absence of robust prognostic indicators, immunological assessment is recommended for all patients at diagnosis.

## TBX1 deficiency

Dominant variants in *TBX1* are a rare monogenic cause of DGS [62–66]. TBX1 is a member of the highly conserved T-box family of transcriptional regulators that play important roles in varied aspects of embryonic development*.* TBX1 controls the expression of approximately 2000 genes through epigenetic modifications [67, 68]. Its expression during embryogenesis requires precise regulation for the coordinated segmentation and development of the structures originating from the 3rd and 4th PPs in a mechanism dependent on the neural crest–derived mesenchyme [69]. Functional interactions between TBX1, CHD7 and retinoic acid (RA) signalling are thought to be responsible for the phenotypic overlap between these three DGS-causing factors [70, 71]. Twelve mutations, both loss- and gain-of-function, have been reported in sporadic and familial cases with clinical features similar to DGS, including CHD, hypoparathyroidism and velopharyngeal insufficiency. Thymic hypoplasia has been observed in at least 3 patients [62, 64]. As mentioned previously, *TBX1* haploinsufficiency in 22q11.2del is associated with more severe immunodeficiency [48]. In the published cohorts of cDGS patients treated with thymus transplantation, only one patient with a suspected pathogenic *TBX1* variant is reported [72]. *TBX1* has 9 exons and 3 isoforms that differ in their use of alternative exon 9 sequences. The reported mutations span exons 3–8, and specifically exon 9C, but not 9A or 9B. There is no clear genotype-phenotype correlation, except for mutations in exon 9C consistently being found in patients without CHD [62, 63, 65]. Variable penetrance is reported, likely because of modifier genes and epigenetic differences influencing *TBX1* expression levels [68]. Detailed immunological assessments are not available.

## TBX2 deficiency

TBX2, another T-box factor, forms a regulatory network with TBX1 and TBX3 that controls the development of pharyngeal apparatus and its derivatives [73]. This may account for the phenotypic overlap between TBX2 and TBX1 deficiency. A variable DGS phenotype with T cell immune deficiency, CHD, hypoparathyroidism and craniofacial dysmorphism has been reported in 3 members of single family with a dominant *TBX2* variant (R20Q) resulting in reduced protein expression and partial loss of transcriptional repressor activity [74]. Other features, also with variable expressivity, included cleft lip/palate, skeletal malformations, endocrinopathies and developmental delay [74]. One of the children had cDGS, treated successfully with thymus transplantation, and the other had pDGS. Their mother’s immune status was first assessed in adulthood, revealing normal total T cell numbers but very low naïve CD4^+^T cells consistent with impaired thymic output [74]. A fourth, unrelated patient, with a distinct heterozygous variant, did not display symptoms suggestive of immunodeficiency [74]. Identification of *TBX2* mutations in more patients with thymic hypoplasia/aplasia will help confirm it as a DGS-causing gene.

## Partial monosomy 10p

Partial monosomy of the short arm of chromosome 10, sometimes called DGS2, is rare having been reported in approximately 50 cases with variable clinical phenotypes, including thymic hypoplasia [75]. Details about the incidence and severity of immunodeficiency in DGS2 are not available. In addition to DGS-like characteristics, other features include sensorineural hearing loss, intellectual disability, craniofacial malformations and growth retardation [76–79]. Mapping of patient mutations has revealed two critical regions on chromosome 10p—HDR1 and DiGeorge Critical Region 2 (DGCR2)—associated with distinct clinical features [80–82]. Haploinsufficiency of the distally located HDR1 region is associated with hypoparathyroidism, deafness and renal anomalies [83], whereas deletion of the more proximal DGCR2 is associated with CHD and thymic hypoplasia [81]. One DGCR2-located gene, *CELF2* (previously called *BRUNOL3*), is a premRNA alternative splicing factor that is strongly expressed in the developing thymus and has been proposed as a candidate gene for thymic hypoplasia [84]. However, pathogenic *CELF2* variants have not yet been reported in patients with thymic hypoplasia.

## CHD7 deficiency and CHARGE syndrome

CHARGE syndrome describes the association of Coloboma, Heart defects, Atresia choanae, Retarded growth/development, Genital and Ear anomalies/deafness [85], occurring in 1 in 15,000–17,000 live births [86]. The underlying molecular cause for up to 90% of cases is haploinsufficiency of *CHD7,* encoding a chromatin-remodeling enzyme that mobilises nucleosomes to regulate the transcription of several developmental transcription factors and pathways [87–89]. It has been postulated that a functional interaction between CHD7, TBX1 and RA signaling could explain the phenotypic overlap in DGS due to CHARGE syndrome, 22q11.2del, TBX1 deficiency, and RA embryopathy [70, 89]. *CHD7* is expressed in the neural crest–derived mesenchyme of the pharyngeal apparatus as well as in TEC, with roles in regulating 3rd PP patterning, thymic organogenesis, *FOXN1* expression and TEC function [69, 90, 91].

Almost all cases are sporadic, with de novo *CHD7* mutations occurring predominantly on the paternal allele [86]. Rare familial cases have been reported due to parental somatic or germline mosaicism and parent-to-child transmission [86]. Over 900 pathogenic *CHD7* variants have been identified*;* nonsense and frameshift mutations predominate over splice site and missense mutations, and there are no obvious hotspots [69, 86]. There is no clear correlation between mutation location and clinical phenotype, particularly as there is variable expressivity in patients carrying recurrent or similar mutations [86]. However, missense mutations are associated with a milder phenotype and less often with CHD, choanal atresia and cleft lip/palate than truncating mutations [86, 89, 92]. In the minority of CHARGE patients in whom *CHD7* mutations are not detected, genetic deficiencies of *CHD7* that are not identifiable using standard techniques may still underlie the disease, including mutations in noncoding regulatory regions, rearrangements and whole gene/exon deletions [86, 89]. Mutations in *SEMA3E* have been described in 2 unrelated patients lacking *CHD7* variants, however neither were reported to have a thymic defect [86, 89]. Finally, it is likely that some patients diagnosed with CHARGE syndrome in fact suffer with distinct, still undefined conditions with overlapping clinical features.

Susceptibility to recurrent otitis media, respiratory and urinary tract infections in CHARGE syndrome may have an anatomical basis rather than being caused by a clinically relevant immune defect [87, 93]. Variable degrees of T cell lymphopaenia are present in 60–80% of patients [93, 94]. For the majority, this appears to be more pronounced in infancy with a tendency to normalise over time [87]. Less frequently, infants may present with athymia and profound T cell lymphopaenia or OS [27, 87]. This is almost always associated with hypocalcaemia, which is not surprising in view of the common developmental origins of the thymus and parathyroids, and thus presents as a cDGS requiring urgent corrective management [27, 87, 94].

## Chromosome 2p11.2 microdeletions

Recently, microdeletions at chromosome 2p11.2 have been reported in five kindreds with variable DGS features, including T cell–specific lymphopaenia and hypocalcaemia, but no CHD [95]. The probands in four of these kindreds were identified through newborn screening (NBS) for SCID. Chromosomal microarray analysis of the probands and their relatives led to the identification of overlapping microdeletions at chromosome 2p11.2 in a total of 13 individuals with variable clinical penetrance. All deletions encompassed the gene *FOXI3*, a FOX gene family member. *FOXI3* haploinsufficiency had previously been described in one patient with aural atresia, velopharyngeal insufficiency and agenesis of the internal carotid artery, suggesting a role in the development of the pharyngeal apparatus [96]. No information is available about this patient’s immunological phenotype. *Foxi3*^-/-^ mouse embryos display impaired PP segmentation [97] and *Foxi3* expression in the pharyngeal apparatus is concomitant to that of *Tbx1* [98]. In the mouse, *Tbx1* and *Foxi3* have been shown to interact genetically, with thymus and parathyroid gland defects observed in *Tbx1*^*+/-*^*Foxi3*^*+/-*^ double heterozygotes [99]. FOXI3 also plays a role in epithelial differentiation in the epidermis and haploinsufficiency has been described in hairless dogs with ectodermal dysplasia [100, 101].

## Nongenetic determinants of DGS

Foetal exposure to RA, maternal diabetes and alcohol are associated with a DGS phenotype [70, 102]. Functional interactions have been identified linking RA metabolism and signalling to both TBX1 and CHD7, and it appears that fine balance of RA homeostasis is required for proper thymic development [70, 103]. In chick embryos, both increased and reduced RA levels result in downregulation of pharyngeal *Tbx1* expression [104]. Similarly, in the mouse, positive and negative perturbations of RA signalling impair thymic development. RA exposure during mouse embryogenesis results in thymic hypoplasia, and altered *Pax1* and *Hoxa3* expression [105]. On the other hand, when RA signaling is abrogated in murine TEC, TEC development and function are impaired [106]. Maternal diabetes was found in about 15% of cases in the first published cohort of cDGS patients considered for thymus transplantation [107]. The mechanisms by which diabetic embryopathy causes DGS may include reduced cytochrome P450-dependent RA catabolism and impaired migration of neural crest–derived mesenchymal cells [108, 109].

## FOXN1 deficiency

FOXN1 is a member of the forkhead/winged-helix family of transcription factors expressed by epithelial cells of the thymus and skin [110, 111]. It is regarded as the master transcriptional regulator for TEC [112]. Although the initial specification of TEC precursors within the thymic anlage occurs independently of FOXN1, it is thereafter indispensable for further TEC development including differentiation of the c- and mTEC lineages, their postnatal maintenance and functional ability to foster thymopoiesis [18, 112–114]. Amongst the genes that have been identified as direct FOXN1 targets in TEC are *CCL25* and *CXCL12*, encoding chemokines that regulate the colonisation of the thymus by lymphoid progenitors; *DLL4*, which is responsible for T lineage commitment of these progenitors; as well as several genes relevant to antigen presentation and therefore thymocyte selection [18]. Outside of the thymus, FOXN1 functions to regulate growth and differentiation of epithelial cells within the epidermis, hair follicles and nail beds [111].

Biallelic mutations causing complete loss of FOXN1 expression and/or function underlie the nude SCID phenotype, named after the association of hairlessness with severe T cell immunodeficiency. Thirty years after its original description in the mouse [115], this phenotype was reported in siblings from Acerno in Italy who presented with T^-^B^+^NK^+^ SCID, congenital alopecia totalis and nail dystrophy, and were found to have a homozygous nonsense mutation in *FOXN1* (R255X) [116, 117]. The same mutation has been found in several other cases, with all but one, originating from Acerno [118, 119]. Two further autosomal recessive mutations have been identified in isolated cases – R320W and S188fs [118, 120]. FOXN1 contains an N-terminal domain (aa 0-269), important for TEC differentiation; followed by the forkhead domain (aa 270-367), which mediates binding to a 5 bp DNA motif within the promoters of its target genes; and a C-terminal domain responsible for activation of target gene transcription (aa 368-648) [121]. R255X and S188fs lead to the introduction of premature stop codons before the DNA recognition motif within the forkhead domain, and regardless are predicted to result in nonsense-mediated decay [116, 122]; R320W directly disrupts the DNA-binding motif necessary for FOXN1’s transcriptional activity [121]. Hence, all nude SCID-associated mutations are thought to lead to a null effect.

Infants with nude-causing mutations present with a typical SCID phenotype with early onset of life-threatening infections. Immunological assessment reveals evidence of defective thymopoiesis, with T cell lymphopaenia that more severely affects the CD4^+^ vs. the CD8^+^ lineage; profound reductions in CD45RA^+^ naïve T cells, CD31^+^ recent thymic emigrants (RTEs) and T cell receptor excision circles (TRECs); impaired T cell proliferation; and absence of thymic tissue on imaging. Although NK and B cell counts are generally normal, the latter are functionally impaired without T cell help. The clinical and immunological features of OS may also be present [117, 118, 120, 122]. Nude SCID requires early definitive management. Unlike for most other forms of SCID, the best treatment for nude SCID is not HSCT, but thymus transplantations which corrects the primary immunological defect and provides the best opportunity for survival [118, 122]. The associated alopecia and nail dystrophy are not corrected by thymus transplantation, reflecting an intrinsic role for FOXN1 in epithelial cells at these sites [17].

Whilst nude SCID is exceedingly rare, with only a handful of cases reported, the inclusion of TREC analysis in NBS programmes has identified a number of patients with hypomorphic compound heterozygous and heterozygous mutations in *FOXN1* [123, 124]. These include heterozygous carriers of nude SCID-associated mutations, as well as a variety of novel, mainly frameshift mutations affecting the forkhead and C-terminal domains [123, 124]. Intriguingly, none have had congenital alopecia, but nail dystrophy is present in approximately half [123, 124]. The spectrum of immune deficiency is variable. Few patients have presented with immune deficiency severe enough to necessitate definitive treatment; HSCT has been used empirically but does not result in T cell reconstitution [123, 124]. More frequently, patients display milder degrees of T lymphopaenia, which are more pronounced in infancy, may be symptomatic or asymptomatic, and tend to improve over time [123, 124]. Taken together, these observations are in support of a gene dosage effect for *FOXN1*, which from the perspective of TEC function is more important in embryonic and early postnatal life, and it is likely that the degree of immune impairment in FOXN1-deficient states correlates with the amount of residual FOXN1 function [123].

## PAX1 deficiency

PAX1 belongs to the paired box family of transcription factors, which play important roles in regulating cell fate determination and body patterning during embryonic development [125]. During embryogenesis, PAX1 is expressed in the sclerotome, from which the vertebral column develops, as well as in all four PPs where it plays a role in regulating pattern formation [125]. Although *PAX1* expression in the 3rd PP initially precedes *FOXN1*, it continues to be expressed in the thymic anlage and later in cTEC in a FOXN1-dependent manner [112].

Autosomal recessive *PAX1* mutations cause a rare syndrome called otofaciocervical syndrome type 2 (OTFCS2) [126, 127]. Clinical features include facial dysmorphism, ear anomalies and hearing impairment, branchial cysts/fistulas, skeletal malformations affecting the vertebrae and shoulder girdle, and mild intellectual impairment [126, 127]. Immunological assessment of 6 patients was consistent with T^-^B^+^NK^+^ SCID [126]. T cells, where present, had a memory phenotype, and there was evidence of absent thymic output with a lack of naïve T cells, RTEs and TRECs, and athymia on imaging; two presented with OS. All 4 patients empirically treated with HSCT failed to reconstitute their naïve T cell compartment, consistent with a thymic stromal cell aetiology for their immunological defect. This was supported by studies showing that the *PAX1* mutations impair its transcriptional ability, likely via altered binding to DNA and that thymic epithelial progenitor cells (TEPCs), differentiated in vitro from patient-derived induced pluripotent stem cells (iPSCs), have reduced expression of several genes involved in PP patterning, and TEC development and function, including *FOXN1* and several of its target genes [126].

## EXTL3 deficiency

Exostosin-like 3 (*EXTL3*) encodes a glycosyltransferase involved in heparan sulphate proteoglycan (HSPG) biosynthesis [128]. Biallelic hypomorphic mutations in *EXTL3* have been described in a rare syndromic immunodeficiency characterised by skeletal dysplasia, and more variably by neurodevelopmental delay and T cell lymphopaenia [128–131]. T lymphopaenia has been described in just over half of the 14 reported cases, ranging in severity from T^-^B^+^NK^+^ SCID +/- OS, to milder deficiencies [128–131]. The pathogenesis of immunodeficiency in *EXTL3* deficiency appears to be multifactorial [129, 131]. HSPGs bind to and modulate morphogenetic growth factors important in both hematopoietic progenitor and thymic stromal cell differentiation; accordingly, patient-derived iPSCs are impaired in their ability to differentiate into both early lymphoid progenitors and TEPCs [129]. HSCT was performed in one case, resulting in correction of the immune defect but not the significant neurodevelopmental and skeletal abnormalities, and there was spontaneous partial recovery of T cell counts and function over time in a further patient, indicating that definitive treatment decisions are not straightforward in this condition [128, 129].

## TTC7A deficiency

Loss-of-function mutations in *TTC7A*, which encodes a tetratricopeptide repeat domain containing protein, have been described in several patients with gastrointestinal tract pathology and primary immune deficiency [132–135]. There appears to be a genotype-phenotype correlation with null mutations causing multiple intestinal atresias and combined immune deficiency, affecting T, B, and NK cell lineages, which may be severe [133]. Hypomorphic mutations, on the other hand, cause early onset inflammatory bowel disease with variable degrees of immune deficiency, autoimmunity, alopecia and nail dystrophy [135]. Study of patient mutations has revealed the role of TTC7A in actin cytoskeleton dynamics important for lymphocyte proliferation, adhesion and migration as well as gut epithelial cell polarity, growth and differentiation [133, 135]. In addition to its expression in cells of the lymphoid lineage and in gut epithelial cells, TTC7A is also expressed in TEC, and a derangement in TEC architecture was observed in a postmortem sample from a patient with TTC7A deficiency [134]. This has raised the possibility that a TEC defect may also contribute to the immunophenotype observed in patients with TTC7A deficiency, although further studies are needed to define the function of TTC7A in TEC and the consequences of its deficiency on TEC development and function [134, 135].

## Defects in lympho-stromal crosstalk

Haematopoietic cell intrinsic defects that severely impair early T cell development also impede TEC differentiation and function due to a lack of crosstalk signals normally provided by developing thymocytes [22]. This has been studied for several forms of SCID in which the primary defect lies within the haematopoietic lineage, including those caused by genetic deficiencies of *RAG1/2*, *AK2*, *IL2RG*, *CD3D*, *ADA*, *RMRP*, and *ZAP70* [22, 136, 137]. Examination of the thymic microenvironment in such patients shows aberrant architecture, TEC differentiation, AIRE expression, reduced DCs and FOXP3^+^ T regulatory cells [22, 136, 137]. The observed deficiencies in mTEC, AIRE expression and thymic T regulatory cells are thought to underlie the propensity for autoimmune disease and OS, particularly in patients with “leaky” SCID, in which very limited residual intrathymic T cell development does occur but in the absence of a thymic microenvironment suitably developed for central tolerance induction [22, 136, 137]. Importantly, thymic stromal cell defects that occur secondary to a lack of thymocyte-derived crosstalk signals are often correctable via treatment of the primary haematopoietic defect, for example with HSCT [138].

## AIRE deficiency

Deficiency of AIRE impairs mTEC function as it affects their ability to properly induce T cell central tolerance [139]. As this is the focus of another article within this issue, we only consider this topic in brief. AIRE is a transcriptional facilitator responsible for the promiscuous expression of thousands of tissue-restricted antigens by mTEC against which the TCRs of developing thymocytes are tested in order to prevent the export of overtly self-reactive T cell clones to the periphery, where they could elicit autoimmune disease [139]. Biallelic mutations in *AIRE* underlie autoimmune polyglandular syndrome 1 type (APS1), also known as autoimmune polyendocrinopathy candidiasis ectodermal dystrophy (APECED) syndrome [140]. APS1 is characterised by early onset multisystem autoimmune disease and the presence of multiple autoantibodies. The classical clinical triad consists of chronic mucocutaneous candidiasis, due to neutralising serum autoantibodies against IL-17 and IL-22, hypoparathyroidism and primary adrenal insufficiency [139, 141]. This presentation is often accompanied by additional autoimmune manifestations, including primary gonadal failure, type 1 diabetes mellitus, pernicious anaemia, thyroid disease and hepatitis, as well as features of ectodermal dystrophy such as tooth enamel hypoplasia and nail dystrophy, which may not have an autoimmune basis [139]. Diagnosis relies on the combination of clinical features, genetics and the detection of typical autoantibodies. Management is symptomatic and may include hormone replacement, antifungals and immunosuppression [139]. Monoallelic, dominant negative mutations within the molecule’s PHD1 (plant homeodomain 1) and SAND (SP100, AIRE, NucP41/P75 and DEAF1) domains have also been described in patients with late presentation, attenuated phenotypes and incomplete penetrance [142–144].

## Diagnosis of inborn errors of thymic stromal cell development

Infants with thymic stromal cell defects that abrogate or severely impair thymopoiesis may present in several ways. Increasingly, patients are identified via population-based NBS for SCID, which relies on the quantification of TRECs in dried blood spots to identify T cell lymphopaenia [145]. NBS is able to identify neonates with the most severe thymic defects (i.e., those associated with SCID, cDGS or OS), as well as those with less critical T lymphopaenia, including at least some cases of pDGS and hypomorphic *FOXN1* deficiency [123, 124, 129, 145]. In the absence of NBS, infants with thymic defects may be identified after clinical presentation with frank immune deficiency +/- OS. In patients who present with a T^-^B^+^NK^+^ SCID, and are treated empirically with HSCT, failure to reconstitute the naïve T cell compartment despite adequate engraftment should raise the possibility of a defective thymic niche for T lymphopoiesis [123, 126]. Patients may also come to the attention of various medical specialties as a result of associated syndromic features. Therefore, a low index of suspicion is required, and there is a case for performing a basic immunological assessment in children with suggestive syndromic features, particularly those consistent with a pharyngeal patterning defect, DGS or CHARGE syndrome.

Thymic output can be assessed by enumerating peripheral blood T cell subpopulations, including naïve CD45RA^+^ T cells and CD31^+^ RTEs, and by measuring TRECs. Thoracic imaging may demonstrate a lack or reduction in visible thymic tissue. The T cell compartment can be further assessed by examining in vitro proliferative capacity and TCR repertoire. A basic immunological workup should also include assessment of B cell function with measurement of serum immunoglobulins and specific antibodies to exposure and vaccination antigens [122].

As the majority of thymic stromal cell defects described have a genetic basis, definitive diagnosis relies on establishing the molecular cause. Clinical features may guide the selection of targeted genetic tests, for example cytogenetic tests such as fluorescence in situ hybridization (FISH) or comparative genomic hybridisation (CGH) where there are features of 22q11.2del, *CHD7* sequencing for CHARGE syndrome, and *FOXN1* for nude SCID. If the clinical presentation is less indicative of a specific molecular cause, broader next-generation approaches can be used including panel-based sequencing, whole exome or genome sequencing.

In the absence of characteristic syndromic features and/or a definitive genetic diagnosis, the identification of a thymic stromal defect is not straightforward. T cell lymphopaenia may mistakenly be ascribed to an undefined haematopoietic defect and a thymic aetiology only suspected after absent immune reconstitution post-HSCT. Novel tools are emerging to facilitate the distinction of primary haematopoietic defects from inborn errors of thymic stromal cells in patients presenting with genetically undefined severe T cell lymphopaenia [126, 129, 146, 147]. These include the use of artificial thymic organoids that express DLL4 and can therefore support the differentiation of CD34^+^ haematopoietic stem cells (HSCs) into mature T cells [146, 147]. In principle, HSCs from patients with thymic stromal cell defects should be able to generate mature T cells, whereas the T cell differentiation capacity of HSCs derived from patients with haematopoietic defects is generally expected to be impaired, although there may be exceptions as has been seen for adenosine deaminase deficiency [146, 147]. Targeted differentiation of patient-derived iPSCs into early lymphoid progenitors or TEPCs has also been used to localise the defect in unclassified T^-^B^+^NK^+^SCID [126, 129]. Finally, reporter assays are available for the functional validation of novel variants in transcriptional factors such as *FOXN1* and *PAX1* [18, 126]. These tools are only available in very specialist centres and comprehensive work is ongoing to validate their use and define their pitfalls, but their possible contribution to the identification of rare patients with undefined T^-^B^+^NK^+^ SCID of thymic origin to whom HSCT should not be offered as a first-line treatment is promising.

## Management of thymic defects including thymus transplantation

Incomplete DGS, most often due to 22q11.2del, is common and requires a multidisciplinary approach. As discussed, thymic hypoplasia in these patients can lead to antibody defects with recurrent infections, autoimmunity and, rarely, malignancy; careful follow-up is thus required. Management guidelines have been published, including special considerations for adults [148, 149].

For the purpose of this review, we will focus on the treatment of congenital athymia, which left untreated is fatal in the first 2 years of life. Prompt initiation of prophylactic measures, including antimicrobials and immunoglobulin replacement, and early referral for corrective treatment are critical for outcome. Allogeneic thymus transplantation is the most appropriate treatment for cDGS [72, 107] and nude SCID [118]. Only two centres, one in the USA and the other in the UK, offer thymus transplantation and have treated more than 100 patients to date. Thymic tissue, necessarily removed during cardiac surgery in infants with normal immunity, is collected and cultured for 2–3 weeks to deplete donor thymocytes before transplantation into the quadriceps muscles of athymic patients. Donor and recipient are blood group compatible, but not matched for major histocompatibility complex (MHC). Bone marrow–derived T cell progenitors of host origin repopulate the transplanted thymic tissue and differentiate into functional T cells, which appear in the peripheral circulation from around 4 months post-transplantation. Recipients acquire a broad T cell repertoire, but counts typically remain below normal. Nevertheless, patients clear infections allowing prophylactic measures to be discontinued. Survival is around 75%, and mortality is mainly associated with pre-existing infections [72, 107]. Despite the absence of MHC matching, long-term tolerance to donor MHC is achieved [150, 151]. The mechanisms supporting positive and negative selection in these MHC-mismatched grafts remain unresolved. Tolerance to most self-antigens is also acquired; however autoimmune complications develop in approximately one-third, mainly causing thyroiditis and autoimmune cytopaenias [72, 107].

Allogeneic thymus transplantation is lifesaving in congenital athymia, but common delays in the diagnosis and/or procedure can significantly complicate treatment and compromise outcome. For instance, atypical features with expansion of autoreactive T cells of memory phenotype are common over time causing a clinical picture of OS [26]. These patients paradoxically require immunosuppression prior to thymus transplantation and the addition of serotherapy with antithymocyte globulin to the transplantation protocol [72]. Delays also increase the risk of infections, which can trigger serious inflammatory complications post-transplantation during immune reconstitution. This is particularly the case for pre-existing viral infections. Given that immune reconstitution post-thymus transplantation is relatively slow, it may even be preferable to proceed with HSCT in the presence of severe viral infection if a matched sibling donor (MSD) is available [152]. If thymus transplantation is accessible, HSCT should otherwise not be attempted as a first-line treatment. Survival of cDGS patients after HSCT using a MSD is around 70%, but is significantly lower when using other donors [153]. Therefore, HSCT should ideally not be considered in absence of an MHC-identical family donor. Immune reconstitution post-HSCT is due to the transfer and engraftment of mature, post-thymic donor T cells, which may confer a higher risk of GVHD [153, 154]. As thymic function remains absent, no naïve T cells are produced and the T cell repertoire is restricted, indicating that HSCT does not constitute definitive treatment, unlike thymus transplantation.

The recent identification of novel thymic defects and the availability of in vitro tools to differentiate primary haematopoietic from thymic stromal cell defects is likely to lead to the consideration of a wider group of patients for thymus transplantation [126, 146, 147]. Of the newer defects covered in this review, the failure of T cell reconstitution post-HSCT, together with the impaired generation of TEPCs from patient-derived iPSCs, in patients with OTFCS2 would suggest that PAX1-deficient patients should be considered for thymus transplantation [126]. A minority of patients with hypomorphic *FOXN1* mutations should probably also be considered. Given the greatly variable severity of T cell immunodeficiency in the published patient series, treatment of patients with hypomorphic FOXN1 deficiency will need to be individualised according to the extent of functional impairment and consequent clinical severity, with thymic transplantation indicated only for those at the more severe end of the spectrum [123, 124]. A better understanding of the long-term impact of these different mutations is crucial before formulation of recommendations regarding therapeutic approaches is possible.

## Concluding remarks

Despite their rarity, thymic stromal cell defects, particularly those causing thymic aplasia, are of great clinical significance because of their potential to cause life-threatening congenital immunodeficiency. Early recognition and distinction from primary haematopoietic defects are critical so that corrective thymus transplantation can be undertaken at the earliest opportunity to give the best chance of survival and good quality immune reconstitution. Although relatively few molecularly defined thymic stromal defects have been attributed to human immunodeficiency and/or autoimmunity to date, the increasing implementation of TREC assays for NBS, together with the availability of technologies for genetic diagnosis and tools for the functional characterisation of novel variants, will undoubtedly result in the discovery and treatment of novel thymic stromal cell defects. Ongoing work within the field seeks to streamline diagnostic and treatment pathways and develop novel approaches for thymic reconstitution, including the use of cryopreserved thymic tissue, which has the potential for MHC matching, transplantation of thymic epithelial progenitor cells and bioengineered artificial organoids [155].
